# Exploratory assessment of the clinical significance of the collateral system between the superior mesenteric artery and inferior mesenteric artery in laparoscopic rectal cancer surgery

**DOI:** 10.1186/s12876-026-05199-1

**Published:** 2026-08-07

**Authors:** Zong-Xian Zhao, Run-Dong Yao, Zong-Ju Hu, Yuan Yao, Shu Zhu, Yong-Xiang Li

**Affiliations:** 1 Department of General Surgery, The First Affiliated Hospital of Anhui Medical University, Hefei, Anhui Province 230022 China; 2 Department of Anorectal Surgery, Fuyang City People’s Hospital, Fuyang, Anhui Province 236000 China

**Keywords:** Rectal cancer, Three-dimensional reconstruction, Anastomotic leakage, Left colic artery, Splenic flexure inter-mesenteric anastomosis

## Abstract

**Background:**

Rectal cancer (RC) is a common and lethal malignant tumor, and surgical treatment is essential. The collateral system between the superior mesenteric artery (SMA) and inferior mesenteric artery (IMA) remains controversial, and its potential clinical significance in RC has not been fully clarified. This study aimed to preliminarily explore and classify this collateral system and its clinical roles in laparoscopic RC surgery.

**Methods:**

This single-center retrospective study enrolled patients who underwent laparoscopic radical resection for RC at Fuyang City People’s Hospital from January 2022 to March 2026. Based on arterial-phase contrast-enhanced computed tomography (CT) images, three-dimensional (3D) reconstructions of the SMA, IMA, and splenic flexure inter-mesenteric anastomosis (SF-ImA) were performed using 3D Slicer. Univariate and multivariate logistic regression analyses were conducted to evaluate risk factors for anastomotic leakage (AL). Subgroup analysis was further performed to evaluate the potential clinical value of SF-ImA in laparoscopic RC surgery.

**Results:**

SF-ImA was detected in 60.10% of RC patients, including 13.94% with central mesocolic anastomosis and 46.15% with intermediate mesocolic anastomosis. The tumor location (low vs. high, OR: 13.377; 95% CI: 2.262–259.500, *P* = 0.018), N stage (OR: 1.339; 95% CI: 1.095–14.183, *P* = 0.038), and surgical duration (OR: 1.026; 95% CI: 1.013–1.042, *P* = 0.001) were independent risk factors for AL. Preserving left colic artery (LCA) (OR: 0.206; 95% CI: 0.062–0.611, *P* = 0.006) was an independent protective factor. Subgroup analysis confirmed that LCA preservation exerted a protective effect against AL exclusively in patients without SF-ImA (OR: 0.041, 95% CI: 0.002–0.357, *P* = 0.012). For patients with SF-ImA, LCA preservation may fail to confer a protective effect against AL and was associated with longer operative duration (*P* < 0.05).

**Conclusion:**

SF-ImA can be effectively evaluated and classified via contrast-enhanced CT combined with 3D reconstruction. Preoperative assessment of SF-ImA may assist individualized surgical planning for RC patients.

## Introduction

Worldwide, including China, rectal cancer (RC) remains a prevalent malignant tumor of the digestive tract, characterized by persistently high mortality and disability rates [1–4]. Currently, surgical intervention remains the cornerstone of the clinical management of RC [5]. The safety and efficacy of RC surgery are closely associated with a precise understanding of local anatomical structures, which is crucial for optimizing surgical outcomes [6–8]. However, the collateral system between the superior mesenteric artery (SMA) and the inferior mesenteric artery (IMA) adjacent to the splenic flexure of the mesocolon remains controversial in clinical anatomy, and its potential impact on RC surgery remains unclear [9].

The terminology for the left colonic mesentery’s vasculature is heterogeneous and often confusing, including terms such as “Riolan’s arch,” “meandering mesenteric artery,” and “Villemin’s arch” [10, 11]. Riolan’s arch was a prevalent anatomical term. It refers to an inconstant central collateral arterial arcade forming a direct anastomosis between the SMA and the IMA. It is distinct from the marginal artery of Drummond, which runs along the large bowel wall [10]. Nevertheless, with the progressive improvement and deeper understanding of regional colonic anatomy, the ambiguous and outdated concept of Riolan’s arch has been gradually phased out in modern anatomical research and clinical practice [12]. Notably, both a high-quality systematic review and a large-sample cadaveric anatomical study have consistently identified the following arterial anastomoses at the splenic flexure of the colon: (1) the marginal artery is present in 100% of cases; (2) splenic flexure inter-mesenteric anastomosis (SF-ImA) are observed in approximately 65% of the specimens, including intermediate mesocolic anastomoses and central mesocolic anastomosis [9, 11, 12]. It is generally acknowledged that Riolan’s arch is roughly equivalent to the central mesocolic anastomosis [12]. Marked interindividual heterogeneity in the incidence and anatomical pattern of these collateral vessels complicates surgeons’ efforts to clarify their potential clinical value during RC resection [12–14].

In RC, the choice of IMA ligation level (high vs. low) remains a major challenge for surgeons. Surgeons must carefully balance oncological principles and anastomotic perfusion to formulate individualized surgical strategies [15]. From the perspective of radical oncologic resection, low IMA ligation in patients with advanced RC carries risks of insufficient lymphadenectomy and increased likelihood of tumor recurrence [16]. Nevertheless, a broad consensus has been reached that high ligation, combined with standardized total mesorectal excision (TME) and D3 lymphadenectomy, can ensure adequate oncological radicality [17–19]. The presence or absence of SF-ImA particularly confuses surgeons when deciding between high ligation and low ligation of the IMA. Theoretically, low ligation with left colic artery (LCA) preservation is redundant and prolongs operative time when SF-ImA is present. In contrast, preservation of the LCA serves as an effective approach to maintain blood perfusion of the left colon and reduce the risk of postoperative anastomotic leakage (AL) in the absence of SF-ImA [10]. This core clinical dilemma raises our central hypothesis: SF-ImA status may influence the protective effect of LCA preservation against AL.

With the rapid advancement of computed tomography (CT) imaging and computer-aided three-dimensional (3D) reconstruction technologies in recent years, 3D-CT vascular reconstruction has become an indispensable tool for quantitative evaluation and visual analysis of vascular anatomy [20–22]. Compared with other research tools, this method allows targeted visualization of the region of interest. It guarantees analytical precision, excludes disturbances from superfluous anatomical tissues, and effectively elevates the accuracy of anatomical studies [23]. Clinically validated, it can provide reliable navigation for surgical procedures [24, 25]. In our previous studies, we adopted contrast-enhanced CT 3D reconstruction to investigate the anatomical characteristics of the SMA, IMA, and inferior mesenteric vein (IMV) during colorectal surgery. The relevant research data have been made publicly available to provide a reference for subsequent anatomical studies [26].

In this study, we utilized contrast-enhanced CT combined with 3D reconstruction to identify and classify SF-ImA status. This study aimed to explore whether SF-ImA status alters the protective impact of LCA preservation on AL, filling existing research gaps concerning the clinical utility of preoperative SF-ImA evaluation in RC surgery.

## Materials and methods

### Study population

**Fig. 1:**
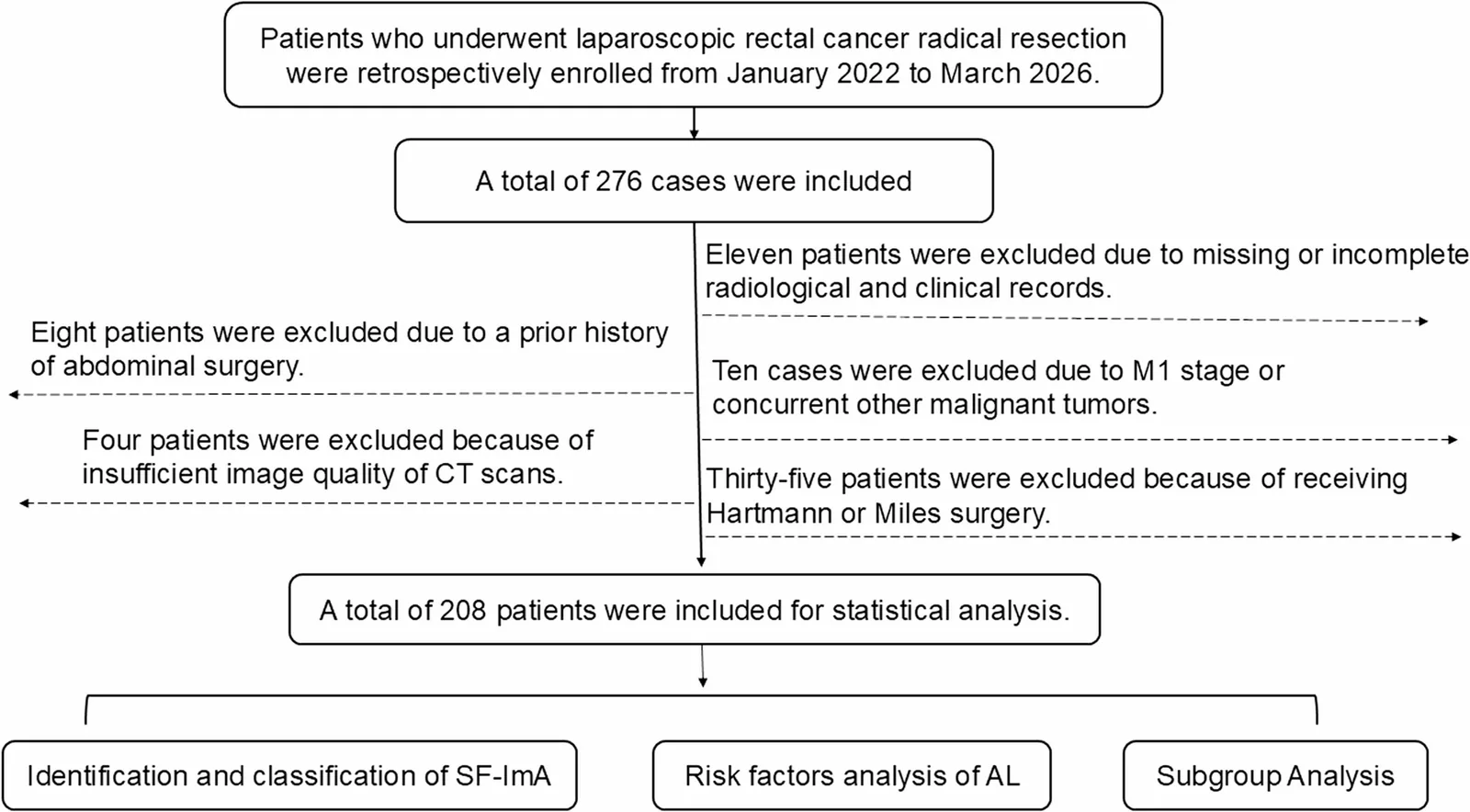
Study design and flowchart. SF-ImA: splenic flexure inter-mesenteric anastomosis; AL: anastomotic leakage

This retrospective study was conducted in the Department of Anorectal Surgery at Fuyang City People’s Hospital from January 2022 to March 2026. The enrollment criteria were: (1) age > 18 and < 90 years; (2) definitive diagnosis of RC confirmed by colonoscopy and pathological examination (adenocarcinoma); (3) receiving laparoscopic radical tumor resection and anastomosis (Dixon); (4) preoperatively performed double-phase abdominal contrast-enhanced CT scan; and (5) written informed consent. Patients were excluded from the study if they met any of the following criteria: (1) presence of metastatic disease (M1 stage); (2) concurrent occurrence of other malignant tumors; (3) decompensated cardiopulmonary dysfunction; (4) cognitive or psychiatric disorders that prevented effective cooperation with treatment; (5) comorbidity with immune system diseases; (6) poor quality of abdominal CT scans that affected image analysis; (7) previous abdominal surgery. This study was approved by the Ethics Committee of Fuyang City People’s Hospital (NO: [2024] 203) and conducted in compliance with the Declaration of Helsinki. All study participants signed an informed consent form, permitting data collection for this study. The study design and flow chart are shown in Fig. 1. Specifically, prophylactic diverting stomas were determined through individualized preoperative assessments. The decision was made after evaluating high-risk factors for anastomotic leakage (neoadjuvant therapy, low tumor location, diabetes, etc.) and fully communicating with patients [27, 28]. LCA preservation was preferentially adopted for patients with early-stage tumors and clear intraoperative anatomy, on the premise of guaranteed radical resection. High IMA ligation was performed at the IMA origin, whereas low ligation was implemented after complete No. 253 lymph node dissection, with ligation distal to the LCA origin. All surgical procedures were performed following the standard principles of TME. Splenic flexure mobilization was performed selectively to achieve tension-free anastomosis. End-to-end colorectal anastomosis was completed with a circular stapler, and intraoperative air leak testing was routinely conducted to verify anastomotic integrity. All operations were completed by the same surgical team with consistent perioperative protocols.

### 3D reconstruction

Enhanced CT examination was conducted as follows: slice thickness 0.625 mm; tube voltage 120 kV; tube current between 250 and 300 mA; and iopromide injection at 1.5 mL/kg, with an injection rate of 3.0–3.5 mL/s. Images were obtained from the arterial phase, which were stored in Digital Imaging and Communications in Medicine (DICOM) format, were imported into 3D Slicer 5.2.2 for reconstruction. In detail, the abdominal aorta, left iliac artery, right iliac artery, IMA, SMA, LCA, sigmoid artery (SA), superior rectal artery (SRA), middle colic artery (MCA), SF-ImA, inferior mesenteric vein (IMV), as well as their associated arteries, veins, and adjacent organs (pancreas), were manually chosen for segmentation and 3D reconstruction. Two radiologists independently evaluated SF-ImA status and subtypes while blinded to surgical details and postoperative outcomes; any discrepant imaging interpretations were resolved via joint consensus review.

### The definition and classification of SF-ImA

**Fig. 2:**
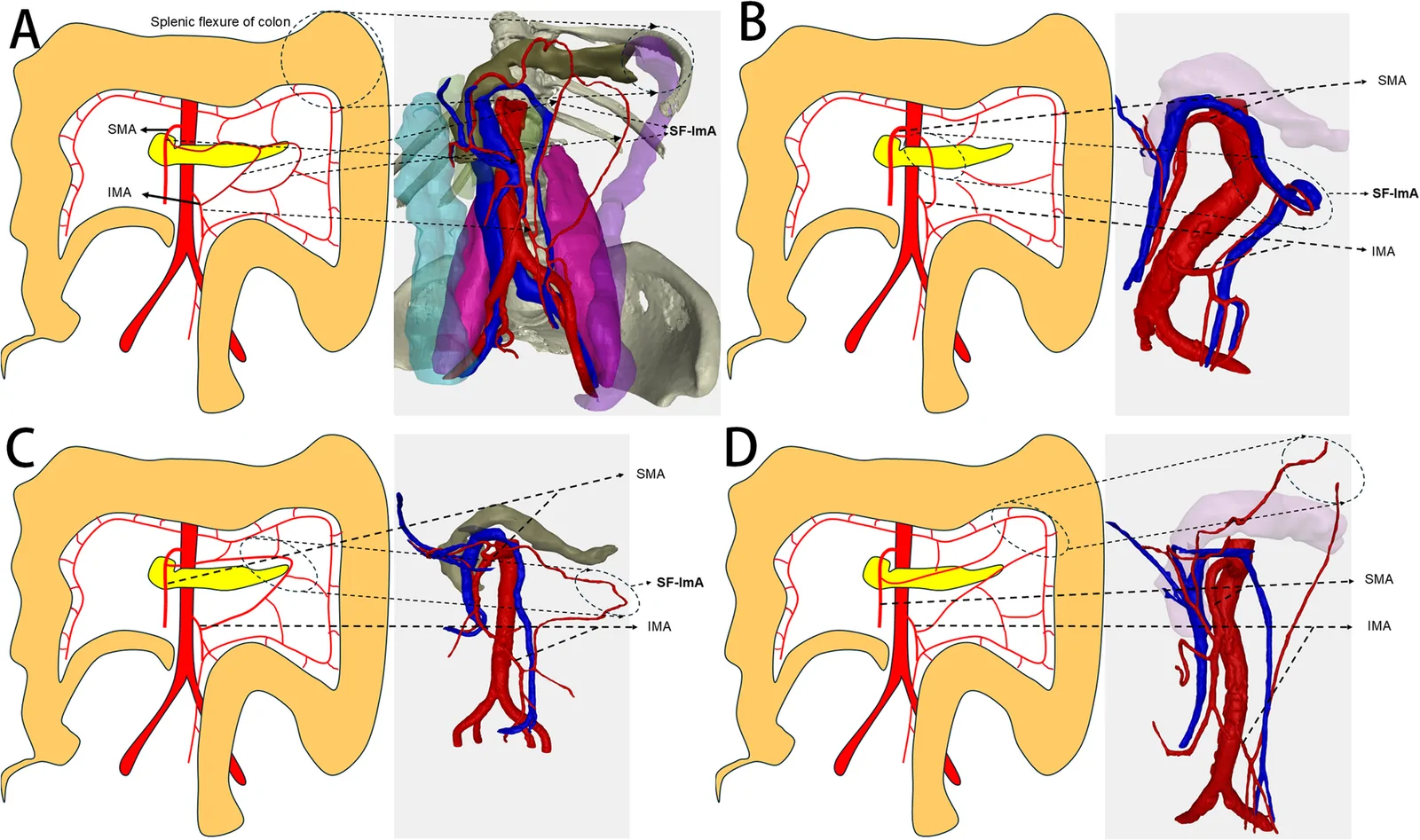
The definition and classification diagram of splenic flexure inter-mesenteric anastomosis (SF-ImA). **A**. SF-ImA is defined as the collateral system between the superior mesenteric artery (SMA) and the inferior mesenteric artery (IMA) at the splenic flexure of the colon. **B**. The central mesocolic anastomosis. **C**. The intermediate mesocolic anastomosis. **D**. The SF-ImA is absent

As shown in Fig. 2, SF-ImA is defined as the mesocolic anastomosis between the SMA and the IMA at the splenic flexure. SF-ImA can be divided into two types: central mesocolic anastomoses and intermediate mesocolic anastomoses. Central mesocolic anastomoses refer to the anastomotic branch close to the main trunks of the SMA and IMA, which courses along the level of the duodenojejunal junction and the inferior margin of the pancreas, lying immediately adjacent to the IMV (Fig. 2B). The intermediate mesocolic anastomosis is located at the pancreatic tail, neither adjacent to the main trunks of the IMA, IMV, nor the duodenojejunal junction (Fig. 2C). In addition, SF-ImA was absent in some cases (Fig. 2D).

### Indices observed

The basic characteristics assessed were: gender, age, body mass index (BMI), clinical stage, tumor location, preservation of LCA, diabetes, hypertension, presence of SF-ImA, operative duration, intraoperative blood loss, number of dissected lymph nodes, anastomotic leakage, and so on. Diagnosis of AL followed internationally accepted unified standards, incorporating clinical manifestations (persistent fever, abdominal pain, peritonitis, and faeculent or purulent output from pelvic drains), radiological evidence (contrast extravasation surrounding the anastomosis), and intraoperative findings during reoperation. AL was categorized using the standard of the International Study Group of Rectal Cancer (ISREC) grading system [29].

### Statistical analysis

Statistical analysis was performed using GraphPad Prism 8 software and R software 4.6.0. For continuous variables, Student’s t-test was applied for normally distributed data with equal variances, Welch’s t-test for normally distributed data with unequal variances, and the Mann–Whitney U test for non-normally distributed continuous variables. Enumeration data were analyzed via the chi-square test or Fisher’s exact test. Logistic regression analysis was applied to identify risk factors. A P value < 0.05 was considered statistically significant.

## Results

### Clinical characteristics

From January 2022 to March 2026, a total of 276 RC patients who received laparoscopic radical resection were initially enrolled (Fig. 1). Eleven patients were excluded due to missing or incomplete radiological and clinical records, and 8 with a history of prior abdominal surgery were also excluded. Four patients were excluded because of insufficient image quality of CT scans, and 10 due to M1 stage or concurrent other malignant tumors. Thirty-five patients were excluded because no bowel anastomosis was performed (Hartmann or Miles procedure). Ultimately, 208 eligible patients (122 male and 86 female) were included for statistical analysis. The baseline clinical characteristics of the study are summarized in Table 1.

**Table 1:** The baseline data of rectal cancer patients

Characteristics	Cases (*n* = 208)
Age (years)	66.93 ± 12.09
Gender	
Female	86 (41.35%)
Male	122 (58.65%)
Height (cm)	163.75 ± 8.43
Weight (kg)	62.37 ± 10.82
Body mass index	23.18 ± 3.12
Body Surface Area (m^2^)	1.64 ± 0.17
Hypertension	
Yes	102 (49.04%)
No	106 (50.94%)
Diabetes	
Yes	67 (32.21%)
No	141 (67.79%)
Serum albumin level (g/L)	43.00 ± 3.73
Location of tumor	
High	56 (26.92%)
Middle	53 (25.48%)
Low	99 (47.59%)
Receiving neoadjuvant therapy	
Yes	78 (37.50%)
No	130 (62.50%)
Surgical duration (min)	194.57 ± 39.02
Intraoperative blood loss (ml)	41.28 ± 33.09
T stage	
T1	26 (12.50%)
T2	41 (19.71%)
T3	60 (28.85%)
T4	81 (38.94%)
N stage	
N0	143 (68.75%)
N1	49 (23.56%)
N2	16 (7.69%)
Number of dissected lymph nodes	16.38 ± 4.43
Lymphovascular invasion	
Yes	54 (25.96%)
No	154 (74.04%)
Nerve invasion	
Yes	28 (13.46%)
No	180 (86.54%)
Preserving LCA	
Yes	117 (56.25%)
No	91 (43.75%)
Prophylactic temporary ileostomy	
Yes	77 (37.02%)
No	131 (62.98%)
Anastomotic leakage	20 (9.62%)
Anastomotic leakage grade	
A	12 (5.77%)
B	8 (3.85%)
C	0 (0.00%)

Tumor location: low rectal (≤ 5 cm), middle rectal (5–10 cm), high rectal (> 10 cm) from the anal verge

*AJCC* American Joint Committee on Cancer, *LCA* left colic artery

### Clinical characteristics analysis of SF-ImA

**Fig. 3:**
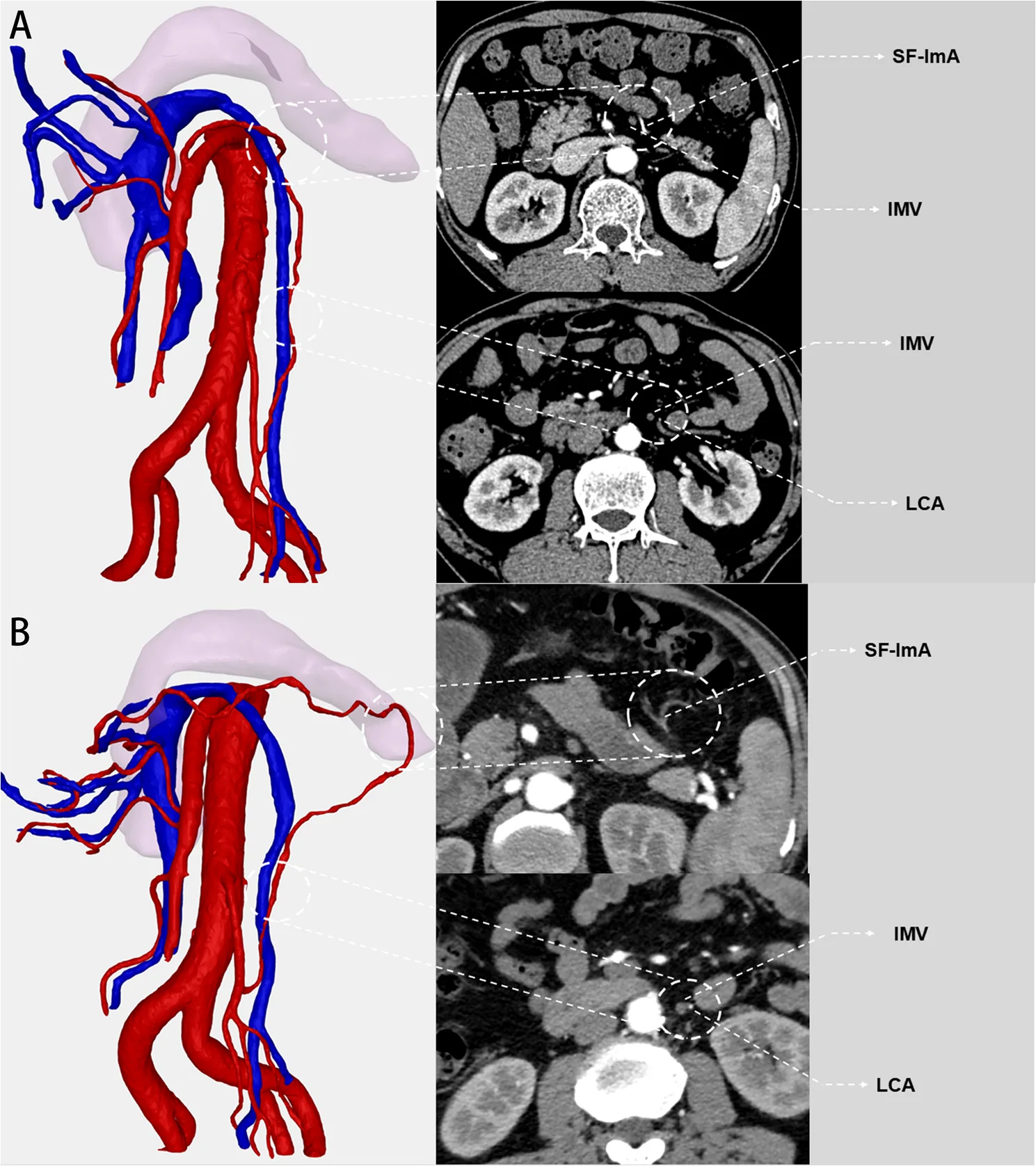
The three-dimensional (3D) reconstruction and enhanced CT manifestation of splenic flexure inter-mesenteric anastomosis (SF-ImA). **A**. The central mesocolic anastomosis. **B.** The intermediate mesocolic anastomosis

We performed 3D reconstruction based on abdominal enhanced CT images of 208 patients and visualized SF-ImA. SF-ImA was identified in 125 cases, accounting for 60.10%. Among these patients, central mesocolic anastomosis was identified in 29 (13.94%, Fig. 3A), while intermediate mesocolic anastomosis was observed in 96 (46.15%, Fig. 3B). To further compare the clinicopathological characteristics between patients with and without SF-ImA, relevant comparative analyses were conducted. As shown in Table 2, there were no statistically significant differences in gender, age, body mass index, diabetes mellitus, tumor location, T stage, N stage, operative duration, AL, and other clinicopathological parameters between the two groups.

**Table 2:** Comparison of clinical characteristics between patients with and without splenic flexure intermesenteric anastomosis (SF-ImA)

Characteristics	With SF-ImA (*n* = 125)	Without SF-ImA (*n* = 83)	t/χ2	*P*-value
Age (years)	66.10 ± 11.39	68.17 ± 12.98	1.204	0.230
Gender			1.813	0.178
Male	78 (62.40%)	44 (53.01%)		
Female	47 (37.60%)	39 (46.99%)		
Height(cm)	164.47 ± 8.04	162.66 ± 8.89	1.516	0.131
Weight(kg)	63.44 ± 10.75	60.75 ± 10.72	1.759	0.080
Body mass index	23.38 ± 3.04	22.89 ± 3.22	1.096	0.274
Hypertension			0.677	0.411
Yes	63 (50.40%)	37 (44.58%)		
No	62 (49.60%)	46 (55.42%)		
Diabetes			0.471	0.493
Yes	38 (30.40%)	29 (34.94%)		
No	87 (69.60%)	54 (65.06%)		
Serum albumin level (g/L)	44.10 ± 3.56	42.85 ± 3.96	0.493	0.623
Location of tumor			4.220	0.121
High	28 (22.40%)	28 (33.73%)		
Middle	31 (24.80%)	22 (26.51%)		
Low	66 (52.80%)	33 (39.76%)		
Receiving neoadjuvant therapy			0.301	0.583
Yes	45 (36.00%)	33 (39.76%)		
No	80 (64.00%)	50 (60.24%)		
Prophylactic temporary ileostomy			0.453	0.501
Yes	47 (37.60%)	30 (36.14%)		
No	78 (62.40%)	53 (63.86%)		
Surgical duration (min)	193.12 ± 41.54	196.83 ± 34.52	0.688	0.492
Intraoperative blood loss (ml)	40.36 ± 35.88	42.77 ± 28.08	0.539	0.590
T stage			2.059	0.151
T1-2	45 (36.00%)	22 (26.51%)		
T3-4	80 (64.00%)	61 (73.49%)		
N stage			0.397	0.529
N0	88 (70.40%)	55 (66.27%)		
N+	37 (29.60%)	28 (33.73%)		
Lymphovascular invasion			1.243	0.265
Yes	29 (23.20%)	25 (30.12%)		
No	96 (76.80%)	58 (69.88%)		
Nerve invasion			0.118	0.732
Yes	16 (12.80%)	12 (14.46%)		
No	109 (87.20%)	71 (85.54%)		
Anastomotic leakage			0.240	0.625
Yes	11 (8.80%)	9 (10.84%)		
No	114 (91.20%)	74 (89.16%)		
Anastomotic leakage grade (*n* = 20)			/	0.999
A	7 (63.64%)	5 (55.56%)		
B/C	4 (36.36%)	4 (44.44)		

#### Risk factors analysis of AL

Clinical and pathological characteristics were compared between patients with and without AL, as shown in Table 3. Patients in the AL group exhibited significantly higher BMI (*P* = 0.021), a greater proportion of comorbid diabetes (*P* = 0.022), higher rates of neoadjuvant therapy administration (*P* = 0.029), prolonged operative time (*P* = 0.009), more advanced nodal disease (*P* = 0.016), and an elevated incidence of lymphovascular invasion (*P* = 0.010). Moreover, patients without LCA preservation were more prevalent in the AL group (*P* = 0.044). No significant differences were observed between the two groups in age, gender, temporary stoma status, or the presence of SF-ImA (all *P* > 0.05).

**Table 3:** Clinical characteristics of patients with and without anastomotic leakage

Characteristics	Anastomotic leakage (*n* = 20)	Non-anastomotic leakage (*n* = 188)	t/χ2	*P*-value
Age (years)	69.90 ± 11.99	66.61 ± 12.06	1.154	0.250
Gender			0.368	0.544
Male	13 (65.00%)	109 (57.98%)		
Female	7 (35.00%)	79 (42.02%)		
Body mass index	24.72 ± 3.80	23.02 ± 2.99	2.333	0.021
Hypertension			1.06	0.302
Yes	12 (60.00%)	90 (47.87%)		
No	8 (40.00%)	98 (52.13%)		
Diabetes			5.263	0.022
Yes	11 (55.00%)	56 (29.79%)		
No	9 (45.00%)	132 (70.21%)		
Serum albumin level (g/L)	42.09 ± 3.20	43.10 ± 3.77	1.156	0.249
Location of tumor			5.536	0.063
High	1 (5.00%)	55 (29.26%)		
Middle	6 (30.00%)	47 (29.26%)		
Low	13 (65.00%)	86 (45.74%)		
Receiving neoadjuvant therapy			4.780	0.029
Yes	12 (60.00%)	66 (35.11%)		
No	8 (40.00%)	122 (64.89%)		
Surgical duration (min)	226.75 ± 52.11	191.22 ± 35.60	2.904	0.009
Intraoperative blood loss (ml)	50.00 ± 25.50	40.40 ± 33.58	1.235	0.218
T stage			1.620	0.203
T1-2	4 (20.00%)	63 (33.51%)		
T3-4	16 (80.00%)	122 (64.89%)		
N stage			5.810	0.016
N0	9 (45.00%)	134 (71.28%)		
N+	11 (55.00%)	54 (28.72%)		
Lymphovascular invasion			6.652	0.010
Yes	10 (50.00%)	44 (23.40%)		
No	10 (50.00%)	144 (76.60%)		
Nerve invasion			2.529	0.112
Yes	5 (25.00%)	23 (12.23%)		
No	15 (75.00%)	165 (87.77%)		
Preserving LCA			4.060	0.044
Yes	7 (35.00%)	110 (58.51%)		
No	13 (65.00%)	78 (41.49%)		
Prophylactic temporary ileostomy			3.068	0.080
Yes	11 (55.00%)	66 (35.11%)		
No	9 (45.00%)	122 (64.89%)		
SF-ImA			0.240	0.625
Yes	11 (55.00%)	114 (60.64%)		
No	9 (45.00%)	74 (39.36%)		

*BMI* body mass index, *SF-ImA* splenic flexure intermesenteric anastomosis, *LCA* left colic artery

Subsequently, univariate and multivariate logistic regression analyses were performed to explore risk factors for AL (Table 4). Univariate analysis identified BMI (OR: 1.180, 95% CI: 1.023–1.367, *P* = 0.024), tumor location (low vs. high, OR: 8.317, 95% CI: 1.588–135.15, *P* = 0.044; middle vs. high, OR: 7.021, 95% CI: 1.144–135.13, *P* = 0.076), diabetes mellitus (OR: 2.881, 95% CI: 1.132–7.520, *P* = 0.027), N stage (OR: 3.033, 95% CI: 1.190–7.925, *P* = 0.020), lymphovascular invasion (OR: 3.273, 95% CI: 1.266–8.481, *P* = 0.013), preservation of the LCA (OR: 0.382, 95% CI: 0.138–0.976, *P* = 0.050), receiving neoadjuvant therapy (OR: 2.773, 95% CI:1.092-7.400, *P* = 0.034), and surgical duration (min) (OR: 1.023, 95% CI: 1.011–1.036, *P* = 0.001) as risk factors. Given the limited number of AL events, only five positive variables were selected for the next multivariate analysis to reduce the risk of overfitting. Multivariate regression further confirmed that tumor location (middle vs. high, OR: 9.862, 95% CI: 1.390-203.827, *P* = 0.049; low vs. high, OR: 13.377, 95% CI: 2.262–259.500, *P* = 0.018), surgical duration (min) (OR: 1.026, 95% CI: 1.013–1.042, *P* = 0.001), lymph node metastasis (OR: 3.815, 95%CI: 1.095–14.183, *P* = 0.038), and preservation of the LCA (OR: 0.206, 95% CI: 0.062–0.611, *P* = 0.006) were independent risk factors for AL.

**Table 4:** Univariate and multivariate logistic regression analysis of risk factors for anastomotic leakage

Characteristics	Univariate logistic regression			*P*-value	Multivariate logistic regression			*P*-value
	β	OR	95% CI		β	OR	95% CI	
Age (years)	0.024	1.025	0.985–1.071	0.250	/			
BMI	0.166	1.180	1.023–1.372	0.024	/			
Location								
high	Ref.				Ref.			
middle	1.949	7.021	1.144-135.138	0.076	2.289	9.862	1.390-203.827	0.049
low	2.118	8.314	1.588-153.149	0.044	2.594	13.377	2.262–259.500	0.018
Albumin (g/L)	-0.075	0.928	0.814–1.052	0.249	/			
Diabetes	1.058	2.881	1.132–7.520	0.027	/			
Neoadjuvant therapy	1.020	2.773	1.092-7.400	0.034	0.093	1.098	0.313–3.815	0.883
T								
T1-2	Ref.				/			
T3-4	0.701	2.016	0.705–7.261	0.227				
N								
N0	Ref.				Ref.			
N+	1.110	3.033	1.190–7.925	0.020	1.339	3.815	1.095–14.183	0.038
Lymphovascular invasion	1.186	3.273	1.266–8.481	0.013	/			
Nerve invasion	0.872	2.391	0.724–6.851	0.121	/			
SF-ImA	-0.232	0.793	0.313–2.056	0.625	/			
Preserving LCA	-0.963	0.382	0.138–0.976	0.050	-1.582	0.206	0.062–0.611	0.006
Temporary stoma	0.815	2.259	0.891–5.872	0.086	/			
Surgical duration (min)	0.023	1.023	1.011–1.036	0.001	0.026	1.026	1.013–1.042	0.001
Intraoperative blood loss (ml)	0.007	1.007	0.994–1.018	0.233	/			

*BMI* body mass index, *SF-ImA* splenic flexure intermesenteric anastomosis, *LCA* left colic artery

### Subgroup analysis stratified by SF-ImA

We further performed subgroup analyses stratified by the presence or absence of SF-ImA, and conducted univariate and multivariate logistic regression analyses to explore risk factors for AL. In the present SF-ImA group, logistic regression analyses revealed that N stage (OR: 5.505, 95% CI: 1.285–27.860, *P* = 0.026) and surgical duration (min) (OR: 1.030, 95% CI: 1.013–1.050, *P* = 0.001) remained independent risk factors for AL. At the same time, LCA preservation (*P* > 0.05) showed no significant association with this complication (Table 5). However, in the absence of the SF-ImA group, tumor location (OR: 22.104, 95% CI: 2.416-653.355, *P* = 0.021) and surgical duration (min) (OR: 1.038, 95% CI: 1.008–1.080, *P* = 0.031) were identified as risk factors (Table 6). Notably, preservation of LCA (OR: 0.041, 95% CI: 0.002–0.357, *P* = 0.012) was protective against AL in this subgroup.

**Table 5:** Univariate and multivariate logistic regression analysis of risk factors for anastomotic leakage in the present SF-ImA group

Characteristics	Univariate logistic regression			*P*-value	Multivariate logistic regression			*P*-value
	β	OR	95% CI		β	OR	95% CI	
BMI	0.1936	1.2136	0.998–1.489	0.053	/			
Location								
High and middle	Ref.				Ref.			
low	0.489	1.631	0.466–6.513	0.454	0.640	1.897	0.426–9.597	0.409
Diabetes	0.716	2.046	0.556–7.251	0.263	/			
Neoadjuvant therapy	1.167	3.213	0.906–11.881	0.069	/			
T								
T1-2	Ref.				/			
T3-4	1.003	2.725	0.664–18.243	0.213				
N								
N0	Ref.				Ref.			
N+	1.167	3.213	0.906–11.881	0.069	1.706	5.505	1.285–27.869	0.026
Lymphovascular invasion	1.139	3.125	0.838–11.257	0.079	/			
Preserving LCA	-0.313	0.731	0.183–2.562	0.633	-1.013	0.363	0.068–1.585	0.196
Surgical duration (min)	0.026	1.026	1.011–1.044	0.001	0.030	1.030	1.013–1.050	0.001

*BMI* body mass index, *SF-ImA* splenic flexure intermesenteric anastomosis, *LCA* left colic artery

**Table 6:** Univariate and multivariate logistic regression analysis of risk factors for anastomotic leakage in the absence SF-ImA group

Characteristics	Univariate logistic regression			*P*-value	Multivariate logistic regression			*P*-value	
	β	OR	95% CI		β	OR	95% CI		
BMI	0.139	1.149	0.930–1.429	0.196	/				
Location									
High and middle	Ref.				Ref.				
low	1.248	3.482	0.847–17.564	0.095	3.096	22.104	2.416-653.355		0.021
Diabetes	1.490	4.435	1.073–22.495	0.047	/				
Neoadjuvant therapy	0.801	2.229	0.545–11.188	0.282	/				
T									
T1-2	Ref.				/				
T3-4	0.260	1.296	0.284–9.192	0.758					
N									
N0	Ref.				Ref.				
N+	0.020	2.772	0.675–12.122	0.155	1.900	6.683	0.962–70.312		0.070
Lymphovascular invasion	1.216	3.375	0.816–14.867	0.091	/				
Preserving LCA	-2.148	0.117	0.022–0.497	0.005	-3.187	0.041	0.002–0.357		0.012
Surgical duration (min)	0.035	1.035	1.012–1.064	0.005	0.037	1.038	1.008–1.080		0.031

*BMI* body mass index, *SF-ImA* splenic flexure intermesenteric anastomosis, *LCA* left colic artery

### Subgroup analysis stratified by LCA preservation

**Fig. 4:**
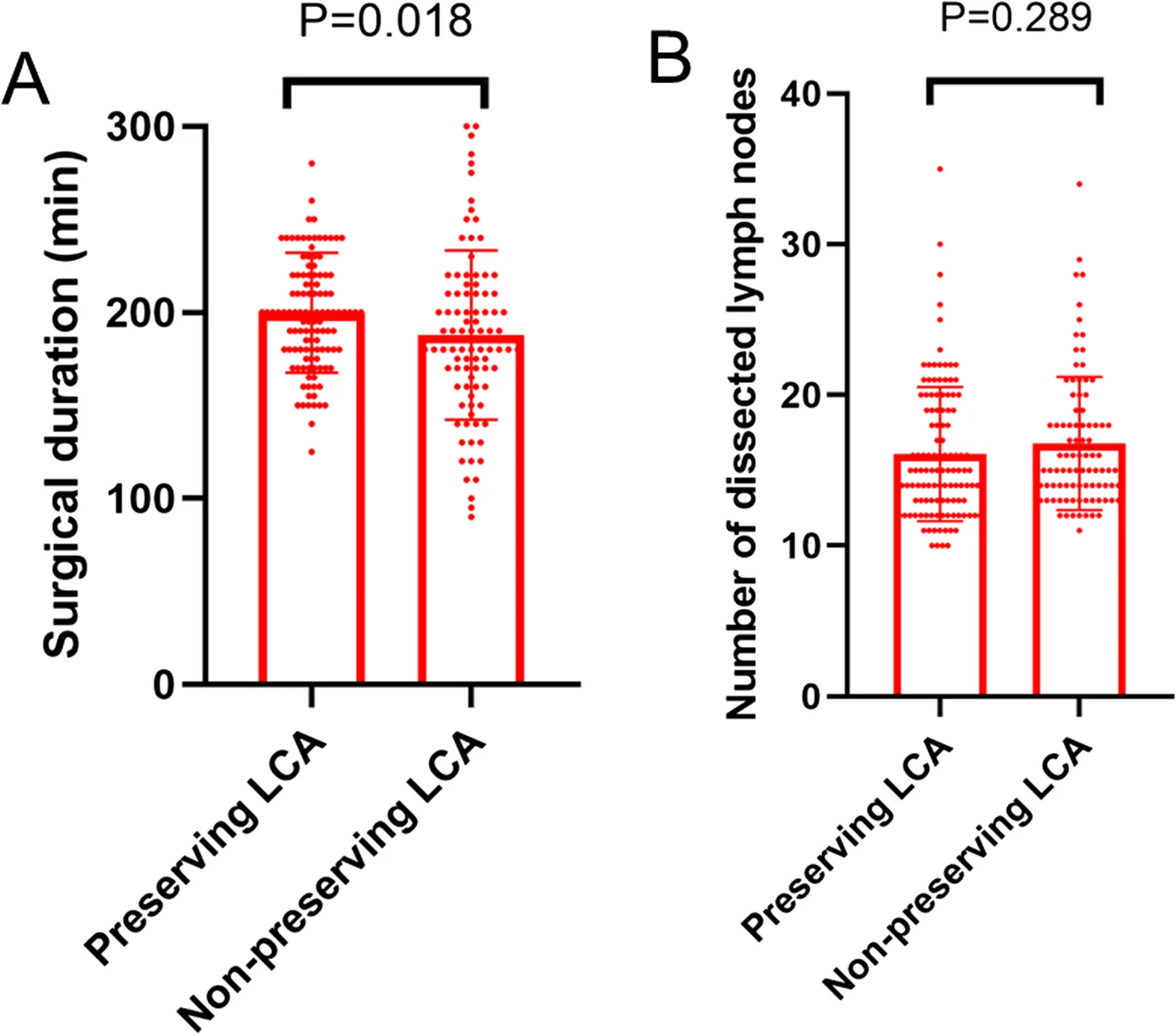
Subgroup analysis stratified by left colic artery (LCA) preservation status. **A**. The operative time was significantly longer in the LCA-non-preservation group (199.87 ± 32.21 vs. 187.91 ± 45.2 min, *P* < 0.05). **B**. No significant difference was found in the number of dissected lymph nodes between the two groups (16.07 ± 4.43 vs. 16.95 ± 4.42, *P* > 0.05)

To further explore the impact of LCA preservation on operative duration and lymph node dissection, we conducted additional subgroup analyses. As shown in Fig. 4, the operative time was significantly longer in the LCA-preservation group (199.87 ± 32.21 min) compared with the LCA-non-preservation group (187.91 ± 45.28 min) (*P* < 0.05). In addition, no significant difference in the number of lymph node dissections was observed between the two groups (preserving LCA vs. non-preserving LCA: 16.07 ± 4.43 vs. 16.95 ± 4.42; *P* > 0.05).

## Discussion

This study performs a preliminary exploration of the clinical significance of collateral vasculature between the SMA and IMA in RC surgery. We first performed 3D reconstruction to visualize the SF-ImA, which could be identified in approximately 60% of patients. Specifically, central mesocolic anastomosis was identified in approximately 15% of cases, and intermediate mesocolic anastomosis in 45%, findings consistent with previous studies [9, 12].

In the multivariate logistic regression analysis, we prudently selected only five variables to reduce the risk of overfitting, considering the limited number of AL events. The covariates included tumor location, neoadjuvant therapy, N stage, LCA preservation status, and operative duration. These five covariates were chosen for their ability to comprehensively capture core clinical characteristics, including tumor staging, anastomotic blood perfusion, and overall surgical difficulty [30–33]. Even with this stringent variable selection strategy, residual confounding from unmeasured factors may still destabilize the regression model, resulting in wide confidence intervals for certain predictors [34]. The results showed that tumor location, advanced N stage, and prolonged operative duration were independent risk factors for AL, whereas the preservation of the LCA was a protective factor. Many studies have indicated that multiple factors jointly contribute to AL following RC resection [17–19]. Tumor location strongly affects the technical difficulty and safety of RC surgery. As reported in most prior studies, patients with low or ultra-low RC who underwent intersphincteric resection (ISR) or transanal total mesorectal excision (TaTME) exhibited a significantly higher risk of postoperative AL than those with middle or high RC [35, 36]. Notably, we were unable to conduct further subgroup stratification for low RC owing to the limited sample size, which may bias our analytical results. Generally, the rectal anastomosis or stump tends to have poor blood perfusion after surgery for low or ultra-low RC. Additionally, anastomotic suturing and reinforcement become technically challenging in low pelvic spaces, and the anastomosis is inevitably subjected to mechanical tension, collectively compromising anastomotic healing and increasing the risk of AL. Besides, advanced tumor stages are frequently accompanied by systemic metabolic disorders, nutritional deficiency, incomplete intestinal obstruction, and anemia [37]. In addition, advanced lesions are often characterized by larger tumor volume, which substantially increases the technical difficulty of the operation. Operative duration is an indirect indicator of surgical difficulty and procedural smoothness. Many studies have confirmed that excessively long surgery markedly raises the incidence of postoperative AL [38–41]. These multifactorial and complicated perioperative conditions collectively impair postoperative tissue repair and physical recovery, thereby independently elevating the risk of AL and adverse surgical outcomes in RC patients [39].

Currently, the ligation level of the IMA remains a major point of controversy in RC surgery, and no consensus has been reached to date [16, 18]. Some studies hold that low ligation with preservation of the LCA can improve blood supply to the rectal anastomosis, reduce the incidence of AL, and exert no adverse impact on oncological outcomes [42–46]. Nevertheless, several investigators hold the opposing view that LCA preservation confers no protective effect against AL, while concurrently extending operative time and increasing intraoperative technical difficulties [7, 47–49]. Moreover, given the considerable individual anatomical variations of the IMA and its branches, the procedure of preserving the LCA can be hampered by complex anatomical structures and may become extremely difficult in certain cases [26]. However, previous studies have neglected the influence of the SF-ImA on the occurrence of AL. From an anatomical perspective, in patients with SF-ImA, the blood perfusion of the left colon can be maintained by blood flow from the SMA via the SF-ImA after high ligation of the IMA. In contrast, for patients without SF-ImA, colonic perfusion after high IMA ligation solely relies on the marginal artery. Given the commonly weak vascular network at the splenic flexure, these patients are at risk of insufficient proximal anastomotic perfusion, which may further induce AL. Furthermore, several studies have reported that elderly patients with atherosclerosis and cardiovascular diseases may experience postoperative systemic hypotension [50–52]. Despite the automatic vascular regulation of regional blood flow, the perfusion pressure of the marginal artery may be inadequate to sustain a normal colonic blood supply under such conditions. For these high-risk patients, LCA preservation appears to be an effective protective surgical strategy.

Subgroup analysis also revealed that LCA preservation slightly prolonged operative duration but had no impact on the quality and extent of lymph node dissection. Consistent with existing meta-analytic evidence, no disparities in lymph node retrieval or oncological efficacy exist between high and low IMA ligation provided that TME and IMA lymph nodal dissection are strictly performed [17, 18]. In addition, previous studies comparing TME and partial mesorectal excision (PME) for proximal RC have shown equivalent long-term oncological outcomes, while less extensive dissection is associated with fewer postoperative complications [53]. Therefore, preoperative evaluation of SF-ImA is critical for stratifying patients by anastomotic perfusion risk, optimizing surgical plans, and reducing operative time. Nevertheless, we do not advocate determining surgical strategies solely based on the presence or absence of SF-ImA. In fact, AL is determined by multifactorial conditions that require comprehensive clinical judgment. Multiple factors, including advanced age, diabetes mellitus, arterial sclerosis, anastomotic tension, and perfusion, jointly affect anastomotic healing. Accordingly, individualized surgical strategies should be formulated based on integrated preoperative evaluation rather than relying on a single anatomical factor. In addition, although our results suggested that LCA preservation had no impact on total lymph node yield, we lacked dedicated data for the apical (station 253) lymph nodes. Therefore, based on the available data, we cannot conclude that different IMA ligation strategies are oncologically equivalent.

Several limitations of this study should be acknowledged. First and importantly, preoperative contrast-enhanced CT was used for the localization and identification of SF-ImA, and the absence of intraoperative confirmation may cause potential deviations. Radiological visualization of SF-ImA merely confirms the existence of this anatomical structure and does not directly translate to adequate perfusion pressure in the distal colon and rectal stump. In addition, owing to the limited sample size and relatively few AL events, we did not perform a formal interaction analysis between SF-ImA status and LCA preservation. The limited number of endpoints, together with confounding factors, also resulted in wide confidence intervals for several predictors, indicating limited estimation precision. Further investigations with expanded sample sizes are needed. Accordingly, the correlative results obtained in our work are preliminary and hypothesis-generating, rather than definitive causal conclusions. Second, the retrospective study design inherently carries risks of bias, and well-designed prospective investigations are required to validate our findings. In the present cohort, clinical decisions to perform a diverting stoma and to preserve the LCA were non-randomized. These confounding factors cannot be eliminated and may compromise the reliability of our results. Third, this study did not incorporate vascular risk factors, including cardiovascular disease, peripheral vascular disease, smoking history, peripheral arterial disease, and atherosclerotic conditions, which may introduce residual confounding in the analysis of AL. Furthermore, quantitative analysis of anastomotic perfusion was not performed. Combination with indocyanine green (ICG) fluorescence imaging is urgently needed in further studies to achieve stratified evaluation of anastomotic blood supply. Finally, this study only analyzed AL and lacked comprehensive evaluation of oncological radicality together with postoperative gastrointestinal, urinary, and sexual function, limiting comprehensive judgment of overall surgical efficacy.

## Conclusion

SF-ImA was identifiable in 60.10% of patients with RC, including central mesocolic anastomosis (13.94%) and intermediate mesocolic anastomosis (46.15%). Lower tumor location, advanced N stage, and prolonged operative duration were independent risk factors for postoperative AL. Preservation of the LCA could significantly reduce the risk of AL in patients without SF-ImA. Preoperative assessment of SF-ImA may assist individualized surgical planning for RC patients.

## Data Availability

The data are available from the corresponding author upon reasonable request.
